# Supplementary material for: Emergent flat band and topological Kondo semimetal driven by orbital-selective correlations
Source: Nat Commun. 2024 Jun 19;15:5242. doi: 10.1038/s41467-024-49306-w (PMC11186837; doi:10.1038/s41467-024-49306-w)
Supplement: Supplementary file 1 — Supplementary Information [file 41467_2024_49306_MOESM1_ESM.pdf]

Supplementary information for: Emergent flat band and topological Kondo semimetal driven by orbital-selective correlations

Lei Chen[1], Fang Xie[1], Shouvik Sur[1], Haoyu Hu[1,2],
Silke Paschen[3,1], Jennifer Cano[4,5], and Qimiao Si[1,*]

[1]Department of Physics and Astronomy, Rice Center for Quantum Materials, Rice University, Houston, Texas 77005, USA

[2]Donostia International Physics Center, P. Manuel de Lardizabal 4, 20018 Donostia-San Sebastian, Spain

[3]Institute of Solid State Physics, Vienna University of Technology, Wiedner Hauptstr. 8-10, 1040 Vienna, Austria

[4]Department of Physics and Astronomy, Stony Brook University, Stony Brook, NY 11794, USA

[5]Center for Computational Quantum Physics, Flatiron Institute, New York, NY 10010, USA

[*]To whom correspondence should be addressed; E-mail: qmsi@rice.edu.

SUPPLEMENTARY NOTE 1: BAND STRUCTURE OF THE ORIGINAL HAMILTONIAN

In this section, we show the dispersion in the original lattice. We fix the reference energy scale $t = 2$. The noninteracting band structure is shown in Fig S1a, in which the bands are clearly separated into the lower two bands and upper three bands. The spectral function is provided in Fig. S1b, where the sharp peak denotes the position of the flat band. We focus on the upper three bands for our Wannier construction.

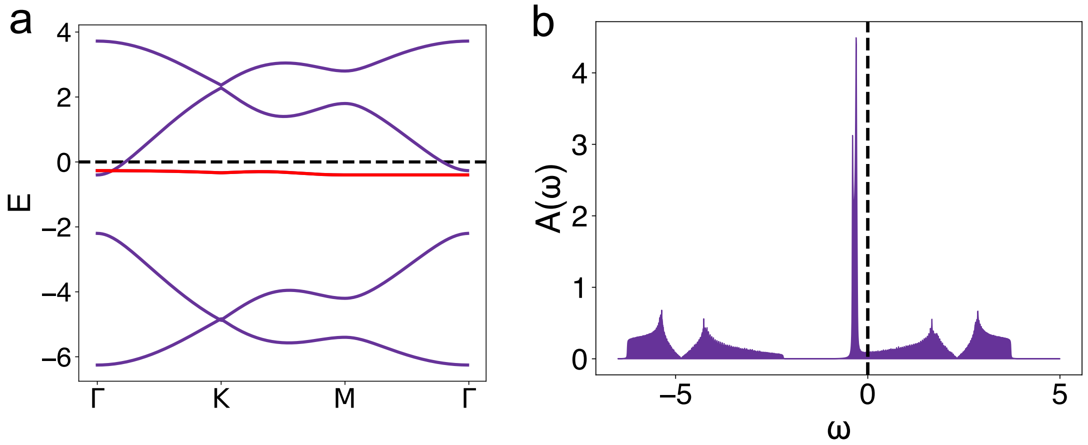

FIG. S1: **The noninteracting electronic structure of the original Hubbard model. a,** The band structure in the original clover lattice, with $\mu = 2.2$, $m = 2$ and $\gamma = -0.2$. **b,** The corresponding density of states.

SUPPLEMENTARY NOTE 2: DESTRUCTIVE INTERFERENCE AND THE ORIGIN OF THE FLAT BAND

In this section, we discuss the origin of the flat band in the clover lattice. Consider the nearest-neighbor hoppings between the blue and yellow sublattices. The kinetic part of the Hamiltonian takes the form as

$$\mathcal{H}_0(\mathbf{k}) = \begin{pmatrix} 0_{2\times2} & \mathcal{H}_{\mathbf{k}} \\ \mathcal{H}_{\mathbf{k}} & 0_{3\times3} \end{pmatrix}, \tag{S1}$$

where $\mathcal{H}_{\mathbf{k}}$ is the hopping matrix between the two groups of sublattices. The localized wavefunction takes the following form[1]:

$$\psi(k_x, k_y) = \begin{pmatrix} 0 & 0 & e^{\frac{k_y}{3}} - e^{-\frac{2}{3}k_y} & e^{-\frac{k_x}{2\sqrt{3}} - \frac{k_y}{6}} - e^{\frac{k_x}{\sqrt{3}} + \frac{k_y}{3}} & e^{\frac{k_x}{2\sqrt{3}} - \frac{k_y}{6}} - e^{-\frac{k_x}{\sqrt{3}} + \frac{k_y}{3}} \end{pmatrix}. \tag{S2}$$

Here, the amplitude is identically zero on the yellow sites because of the destructive interference effect. The real space wavefunction is shown in Fig. S2.

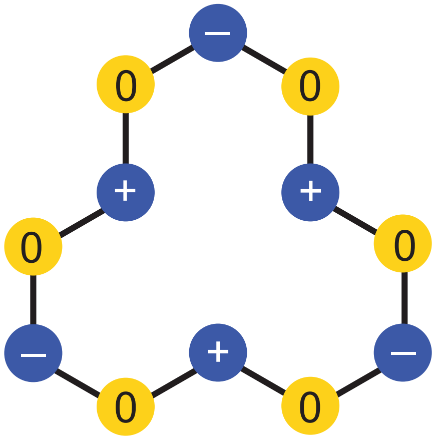

FIG. S2: **Wavefunction for the electronic states of the flat band.** Illustrated here is the real space representation of the electronic wavefunction for the flat band in the clover lattice.

SUPPLEMENTARY NOTE 3: EFFECTIVE MULTI-ORBITAL HUBBARD MODEL

For the kinematic hoppings of the effective molecular orbitals, we refer to Table II in the SM of Ref. 2. However, we focus on the case such that, in the noninteracting case, the flat band lies considerably below the Fermi energy. In Fig. S3, we present the DOS for the d and c electrons in the absence of any hybridization between the two orbitals. For the interaction part, the ratio between the Hubbard interaction of the effective d electron and the one on the original lattice is $u/U = 0.15$[2]. For the same reason that the onsite interaction among the c-orbitals is unimportant, the Hund's coupling does not play an important role here[2].

SUPPLEMENTARY NOTE 4: ANALYSIS OF THE EFFECTIVE MULTI-ORBITAL HUBBARD MODEL WITH A SPIN-ORBIT COUPLING

In this section, we discuss the influence of the spin-orbit coupling (SOC). For our purpose, it is adequate to consider a SOC that is small compared to the other energy scales. We can then analyze the effect of the SOC perturbatively, by using the same Wannier orbitals described above. We thus project the SOC of the original lattice onto the Wannier orbital basis. We consider the SOC along

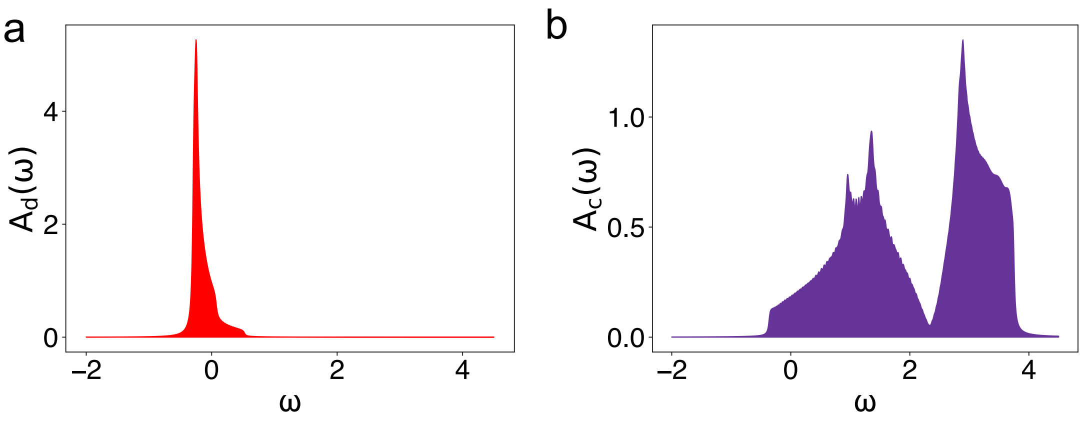

FIG. S3: **The noninteracting density of states projected to the Wannier orbitals. a,b,** The DOS of the d and c electrons, respectively, in the absence of any hybridization between the two orbitals.

the z direction of the original clover lattice, which takes the form as

$$\mathcal{H}_{\rm soc} = \sum_{i,\sigma=\pm} it_{soc}\sigma \left(\eta^{\dagger}_{i\sigma C}\eta_{i\sigma D} + \eta^{\dagger}_{i\sigma D}\eta_{i\sigma E} + \eta^{\dagger}_{i\sigma E}\eta_{i\sigma C}\right) + h.c.\,. \tag{S3}$$

Here, t_{soc} is the strength of the SOC, and $\sigma = \pm$ represents spin up and spin down, respectively. The hopping parameters on the basis of the three Wannier orbitals are shown in Supplementary Table. I and Supplementary Table. II.

Because the value of the SOC is small, we use the same saddle-point parameters as we obtained in the case without the SOC. The renormalization factors Z for the different orbitals will then renormalize the SOC accordingly.

As shown in Fig. S4, the SOC generically gaps out the Dirac nodes (located along $\Gamma - K$) for our 2D model, both for the cases with and without interactions. This leads to flat Z_2 topological bands. We stress that, the flatness of the emergent bands close to the Fermi energy is robust against the SOC.

SUPPLEMENTARY NOTE 5: RESIDUAL INTERACTIONS AND THEIR COMPETITION

We have so far considered the dominant terms of the interactions that are projected into the Wannier basis: the on-site Coulomb interaction among the most localized molecular (Wannier) orbitals (d) and the other on-site interactions between the d orbitals and those of the more extended molecular orbitals (c_1 and c_2), as described by Eq. 1 in the main text. Taking into account these

(i,j)	$(0,1)$	$(-1,0)$	$(1,0)$	$(0,-1)$
$t_{ia_1+ja_2}$	$-0.04566+i0.01088$	$-0.04566-i0.01088$	$-0.04566+i0.01088$	$-0.04566-i0.01088$
	$\pm(2,-2)$	(0, -2)	(0, 2)	(2,0)
	0.04991	$0.03701-i0.0031$	$0.03701+i0.0031$	$0.03701+i0.0031$
	$(-2,0)$	(-1, 2)	(1,2)	(-2,1)
	$0.03701-i0.0031$	$-0.03314-i0.00061$	$-0.03314+i0.00061$	$-0.03314+i0.00061$
	$(2,1)$	$\pm$(1, -1)		
	$-0.03314-i0.00061$	0.02032		
(i,j)	$(1,0)$	$(0,-1)$	$(-1,2)$	$(-2,1)$
$V^1_{ia_1+ja_2}$	$0.13301+i0.01893$	$-0.13301+i0.01893$	$0.09264-i0.00299$	$-0.09264-i0.00299$
	$(1,-2)$	$(2,-1)$	$(0,-2)$	$(2,0)$
	$0.07903-i0.00749$	$-0.07903-i0.00749$	$-0.06361+i0.00909$	$0.06361+i0.00909$
	$(-2,3)$	$(-3,2)$	$(1,1)$	$(-1,-1)$
	$0.05494-i0.00431$	$-0.05494-i0.00431$	$-0.05082-i0.007$	$0.05082-i0.007$
(i,j)	$(1,-1)$	$(-1,1)$	$(0,1)$	$(-1,0)$
$V^2_{ia_1+ja_2}$	-0.15053	-0.14577	$0.096-i0.00896$	$0.096+i0.00896$
	$(0,-1)$	$(1,0)$	$(0,0)$	$(2,-2)$
	$-0.09143+i0.01766$	$-0.09143-i0.01766$	-0.07918	0.07563
	$(1,1)$	$(-1,-1)$	$(-1,2)$	$(-2,1)$
	$-0.07542+i0.00449$	$-0.07542-i0.00449$	$0.07311+i0.00509$	$0.07311-i0.00509$

Supplementary Table I: **Parameters for the noninteracting Hamiltonian in the Wannier basis – part I.** Shown here are the hopping parameters for the d orbitals and the hybridization between the d and c orbitals for spin up when $t_{\rm soc}=0.06$. The parameters in the spin-down case are the Hermitian conjugate of their spin-up counterparts.

interactions at the saddle-point level of the effective Hamiltonian in the slave-spin approach leads to the emergent flat band, in which the (renormalized) electronic quasiparticle states are primarily associated with the effective d orbitals.

The residual interactions for the emergent flat bands are mainly of three types, all of which are primarily pertinent to the d electrons (as opposed to the c_1 and c_2 electrons). The first type is the

(i,j)	$(0,-1)$	$(0,1)$	$(1,0)$	$(-1,0)$
$t^{11}_{ia_1+ja_2}$	$-0.35388+i0.01913$	$-0.35388-i0.01913$	$-0.35388-i0.01913$	$-0.35388+i0.01913$
	$\pm(1,-1)$	$\pm(2,-2)$	(-1,-1)	(1,1)
	-0.15797	-0.08055	$-0.07438+i0.00105$	$-0.07438-i0.00105$
(i,j)	$(0,-1)$	$(0,1)$	$(1,0)$	$(-1,0)$
$t^{22}_{ia_1+ja_2}$	$0.27789-i0.00826$	$0.27789+i0.00826$	$0.27789+i0.00826$	$0.27789-i0.00826$
	$(0,-2)$	$(0,2)$	$(-2,0)$	$(2,0)$
	$-0.07658+i0.0008$	$-0.07658-i0.0008$	$-0.07658+i0.0008$	$-0.07658-i0.0008$
	$(-1,-1)$	$(1,1)$		
	$0.05848+i0.0014$	$0.05848-i0.0014$		
(i,j)	$(0,-1)$	$(1,0)$	$(1,-2)$	$(2,-1)$
$t^{12}_{ia_1+ja_2}$	$0.43550-i0.00395$	$-0.43550-i0.00395$	$0.1959-i0.00079$	$-0.1959-i0.00079$
	$(0,1)$	$(-1,0)$	$(2,-3)$	$(3,-2)$
	$0.15083-i0.00138$	$-0.15083-i0.00138$	$0.11537-i0.00017$	$-0.11537-i0.000179$
	$(0,2)$	$(-2,0)$	$(-1,2)$	$(-2,1)$
	$0.09257+i0.00133$	$-0.09257+i0.00133$	$0.09107+i0.00516$	$-0.09107+i0.00516$
	μ	Δ_1	Δ_2	
	0.15	1.81	2.708	

Supplementary Table II: **Parameters for the noninteracting Hamiltonian in the Wannier basis – part II.** Shown here are the hopping parameters between the c electrons for spin up when $t_{\text{soc}} = 0.06$, the chemical potential μ and the effective crystal field differences Δ_α. The parameters for the spin-down case are the Hermitian conjugate of their spin-up counterparts.

Ruderman–Kittel–Kasuya–Yosida (RKKY) interaction between the d electrons, mediated by the conduction c electrons. For the generic case with typical fillings of the conduction c electrons, the RKKY interaction tends to be antiferromagnetic. However, when the c electrons are dilute, the RKKY interaction would be ferromagnetic.

The second type is the superexchange interaction between the d electrons. This arises because, in the effective (projected) model, the hybridization between the d orbitals in different unit cells is non-zero and so is the hybridization between the d and c orbitals. These kinetic terms induce a cou-

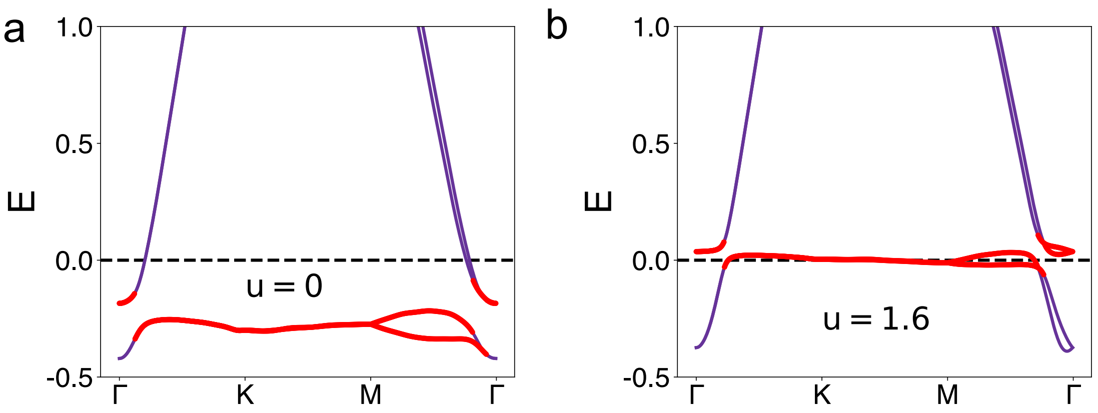

FIG. S4: **The electronic dispersion in the presence of the SOC.** Shown here are the electronic dispersions for $u = 0$ (**a**) and for $u = 1.6$ (**b**), with the SOC in the original lattice to have a strength of $t_{soc} = 0.06$.

pling between the low-energy renormalized (i.e., coherent) part of the d-electron spectral weight near the Fermi energy and the incoherent high-energy part of the d-electron spectral weight (which is controlled by both the u-interaction and bare d-level); when the latter are integrated out, the superexchange interaction ensues. The superexchange interaction is typically antiferromagnetic and is maximized near the (orbital-selective) Mott transition point. Deep in a Mott insulating phase, the value of the superexchange interaction can be derived using a direct perturbation theory in terms of the d-d hopping and d-c hybridization. In the bad metal region, i.e. in the regime of our interest–where the coherent part of the d-electron spectral weight is nonzero but small, such that the system is in proximity to the orbital-selective Mott transition – the superexchange interaction can be constructed by considering the fluctuations beyond the saddle-point level such that the incoherent part of the slave-spin spectral weight is taken into account: This method is in parallel to the derivation for the superexchange interaction in a closely-related slave-particle representation, viz. the slave-rotor approach (see Fig. 2b of Ref. 3).

The third type of residual interactions consists of the direct exchange among the d-electrons. They can be constructed from the projection of the original Hubbard interaction to the Wannier basis. Although the emergent molecular orbitals are exponentially localized, even the most compact (d) orbital still extends over several lattice sites. The shared sites between the nearby molecular orbitals contribute to this ferromagnetic direct exchange interaction. These typically ferromagnetic interactions between the neighboring sites have the form of the Hund's-like two-body interactions.

We have so far focused on spin exchange interactions. The processes leading to the first and third types of residual interactions naturally lead to density-density interactions between neighboring sites as well.

In summary, the forms of the residual interactions are of the spin-spin and density-density interactions among neighboring sites. An appropriate study of the dominant residual interactions should include the above terms, and the competition between them could lead to different phases. For example, if the antiferromagnetic interactions prevail over the ferromagnetic terms, they promote antiferromagnetic-ordering tendencies and the associated quantum criticality. If the ferromagnetic interactions dominate, they may split the Kramers degeneracy, leading to bands with a non-zero Chern number. With the help of other short-range interactions, including the short-range density-density interactions, this regime has the potential to yield fractional Chern insulating (FCI) states.

SUPPLEMENTARY NOTE 6: SLAVE SPIN METHOD

In this section, we derive the slave spin equations we used in the main text. In the slave spin method, the creation operator for the correlated orbital is re-expressed as the product of a SS operator and an auxiliary fermionic operator: $d_\sigma^\dagger = f_{i\sigma}^\dagger o_{i\sigma}^\dagger$, where $o_{i\sigma}^\dagger = P_{i\sigma}^+ S_{i\sigma}^+ P_{i\sigma}^-$ and $P_{i\sigma}^\pm = \frac{1}{\sqrt{1/2 \pm S_{i\sigma}^z}}$. To remove the unphysical Hilbert space, the SS and the auxiliary fermion need to follow the constraint:

$$S_{i\sigma}^z + \frac{1}{2} = n_{i\sigma}^f. \tag{S4}$$

The interaction term can be expressed by the SS operators as described by Eq. 6 in the main text. The noninteracting part of the Hamiltonian now takes the following form:

$$\begin{aligned} H_0 = &\sum_{ij\sigma} t_{ij} \left(f_{i\sigma}^\dagger o_{i\sigma}^\dagger o_{j\sigma} f_{j\sigma} + h.c. \right) - \sum_i \mu f_{i\sigma}^\dagger f_{i\sigma} \\ &+ \sum_{ij,\alpha\sigma} V_{ij}^\alpha \left(f_{i\sigma}^\dagger o_{i\sigma}^\dagger c_{j\alpha\sigma} + h.c. \right) \\ &+ \sum_{ij,\alpha\beta,\sigma} t_{ij}^{\alpha\beta} \left(c_{i\alpha\sigma}^\dagger c_{j\beta} + h.c. \right) + \sum_{i\alpha\sigma} (\Delta_\alpha - \mu) c_{i\alpha\sigma}^\dagger c_{i\alpha\sigma} \,. \end{aligned} \tag{S5}$$

We decouple the f and o at the saddle-point level with $f_{i\sigma}^\dagger o_{i\sigma}^\dagger o_{j\sigma} f_{j\sigma} = f_{i\sigma}^\dagger f_{j\sigma} \langle o_{i\sigma}^\dagger o_{j\sigma} \rangle + \langle f_{i\sigma}^\dagger f_{j\sigma} \rangle o_{i\sigma}^\dagger o_{j\sigma} + const.$ and $f_{i\sigma}^\dagger o_{i\sigma}^\dagger c_{j\alpha\sigma} = \langle f_{i\sigma}^\dagger c_{j\alpha\sigma} \rangle o_{i\sigma}^\dagger + \langle o_{i\sigma}^\dagger \rangle f_{i\sigma}^\dagger c_{j\alpha\sigma} + const.$ We take the single site decoupling

$\langle o_i o_j^\dagger \rangle \approx \langle o_i \rangle \langle o_j^\dagger \rangle$, and Taylor expand $o_{i\sigma}^\dagger$ at the saddle point as follows:

$$
\begin{aligned}
o_\sigma^\dagger &\approx \langle P_\sigma^+ \rangle S^+ \langle P_\sigma^- \rangle + \langle P_\sigma^+ \rangle \langle S_\sigma^+ \rangle \langle P_\sigma^- \rangle \frac{1}{2} \left(S_\sigma^z - \langle S_\sigma^z \rangle \right) \left(\frac{-1}{n_\sigma} + \frac{1}{1-n_\sigma} \right) \\
&= O_\sigma^\dagger + \langle O_\sigma^\dagger \rangle \frac{1}{2} \frac{n_\sigma - 1/2}{(1-n_\sigma) n_\sigma} \left(2S_\sigma^z - (2n_\sigma - 1) \right) \\
&= O_\sigma^\dagger + \langle O_\sigma^\dagger \rangle \eta_\sigma \left[2S_\sigma^z - (2n_\sigma - 1) \right] ,
\end{aligned}
\tag{S6}
$$

where

$$
\begin{aligned}
O_\sigma^\dagger &= \langle P_\sigma^+ \rangle S_\sigma^+ \langle P_\sigma^- \rangle \\
\eta_\sigma &= \frac{1}{2} \frac{n_\sigma - 1/2}{(1-n_\sigma) n_\sigma} .
\end{aligned}
\tag{S7}
$$

We notice that $\langle o_\sigma \rangle = \langle O_\sigma \rangle$. Combining the above equations and introducing the Lagrangian multipler λ_σ to enforce the constraint in Eq. S4, we obtain the self-consistent equations as described in Eq. 5 of the main text.

The Green's function of the d electron is obtained by the convolution of the SS and the auxiliary fermion propagators, with

$$
G(\mathbf{k}, i\omega_n) = \sum_m G_S(i\Omega_m) G_f(\mathbf{k}, i\omega_n - i\Omega_m) . \tag{S8}
$$

After an analytical continuation, we have the spectral function

$$
\begin{aligned}
A_d(\mathbf{k}, \omega) = \frac{2\pi}{Z} \sum_\sigma \sum_{n,m} &\langle n | o_\sigma | m \rangle \langle m | o_\sigma^\dagger | n \rangle \delta(\omega - E_{nm} - \epsilon_{\lambda\mathbf{k}}) |\Lambda_{\lambda\mathbf{k}}^f|^2 \\
&\times \left[e^{-\beta E_m} (1 - f_{\lambda\mathbf{k}}) + e^{-\beta E_n} f_{\lambda\mathbf{k}} \right] ,
\end{aligned}
\tag{S9}
$$

where λ labels the bands solved from H^f, and $\Lambda_{\lambda\mathbf{k}}^f$ represents the corresponding f-component in the eigenvectors. In addition, n, m denote the eigenvectors of H^S, and $E_{nm} = E_n - E_m$ is the energy difference between the two slave spin states. Finally, $f_{\lambda\mathbf{k}} = \frac{1}{e^{\beta\epsilon_{\lambda\mathbf{k}}}+1}$ is the Fermi-Dirac distribution function.

SUPPLEMENTARY NOTE 7: EVOLUTION OF THE SPECTRAL FUNCTIONS ACROSS THE ORBITAL-SELECTIVE MOTT TRANSITION

In this section, we provide further details on the evolution of the single-electron spectral weight when the interactions further increase from the case of $u = 1.6$ shown in Fig. 2b of the main text, approaching and into the OSMP. The evolution is primarily seen in the d-electron DOS (red lines). The c-electron DOS (marked in purple) is not markedly changed.

Fig. S5a shows the DOS for $u = 2.2$, which is smaller than but close to the critical value ($u_c = 2.4$) for the orbital-selective Mott transition. Compared to the case of $u = 1.6$ shown in Fig. 2b of the main text, there is a significant transfer of the d-electron spectral weight from the coherent part (the central red peak near the Fermi energy) to the incoherent part (the side peaks marked by the red dashed lines).

Fig. S5b displays the DOS for $u = 2.5$, where the system is in the OSMP (at $u > u_c = 2.4$). Here, for the d-electron DOS, the coherent peak has vanished. What is left is the incoherent part that comprises the lower and upper Hubbard bands marked by the red dashed lines.

The slave spin method incorporates the nonlocal spin correlations in the system[4], which is important in capturing the orbital-selective Mott transition. The result here has some similarities with its counterpart arising in the context of the Fe-based superconductors[5,6]. There is however one important distinction: the existence of the flat band in the noninteracting dispersion means that the difference between the bandwidths ($D_{\rm flat}$ and $D_{\rm wide}$) is especially large here.

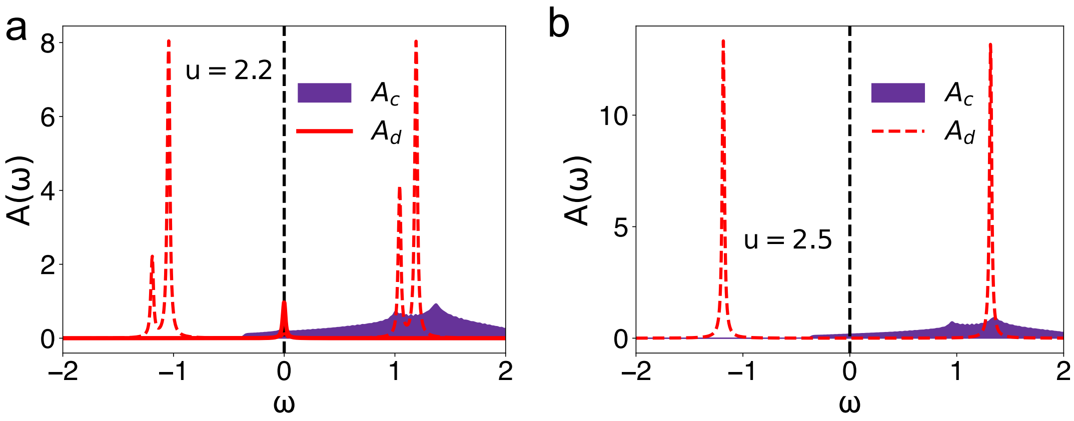

FIG. S5: **Evolution of the DOS across the orbital-selective Mott transition. a,** The DOS of the d and c electrons for $u = 2.2$, which is just below the critical value $u_c = 2.4$. The coherent part of the d-electron spectrum corresponds to the central peak (the red solid line), and its incoherent part appears as side peaks (the red dashed lines). The c-electron spectral function is shown in purple. **b,** The case of $u = 2.5$, which is larger than u_c and, thus, the system lies inside the OSMP. The d-electrons are in a Mott insulating state, and their spectrum contains only the lower and upper Hubbard bands.

SUPPLEMENTARY NOTE 8: PHASE DIAGRAM OF THE EFFECTIVE MULTI-ORBITAL MODEL

In this section, we discuss the overall phase diagram in terms of u and the bare local energy level of the d electron (ϵ_d^0). According to the kinetic Hamiltonian as described by Eq. 4 in the Methods, $\epsilon_d^0 = -\mu$. As ϵ_d^0 decreases, the flat band further deviates from the Fermi energy. We scan the phase diagram as a function of both ϵ_d^0 and the interaction strength u. The curves of quasiparticle weight versus u for the d electron are shown in Fig. S6a for different values of ϵ_d^0, accompanied by the corresponding colormap shown in Fig. S6b. The phase diagram can be divided into three regimes separated by the black dashed line, as depicted in Fig. S6b. In the dark blue region (region "I"), the quasiparticle weight of the d electron completely vanishes, leading to the full localization of the d orbital. In the bright yellow region (region "III"), the quasiparticle weight of the d electron almost equals 1, and the flat band remains distant from the Fermi energy. The intermediate region (region "II"), marked as gradually changing shades of green, corresponds to the orbital-selective correlation region, where we realize an emergent flat band pinned to the Fermi energy. This can be further elucidated by comparing the single-particle excitations with a fixed ϵ_d^0 and varying the interaction strengths u. The dispersions displayed in Fig. S6c-e correspond to the parameter settings marked with purple dots, as shown in Fig. S6b, with $\epsilon_d^0 = -0.15$ and $u = 0.1$, 1.1 and 1.5, respectively. These correspond to the weakly interacting region, mixed-valence region, and the Kondo limit. The dispersions with a local energy further away from the Fermi energy ($\epsilon_d^0 = -0.5$) is shown in Fig. S6f-h, displaying a similar trend.

SUPPLEMENTARY NOTE 9: ORBITAL SELECTIVE CORRELATIONS IN A GENERAL SETTING

In this section, we illustrate that the conclusion we have reached is applicable to other types of geometry-induced flat band systems. (For an overall discussion, see the main text.) The example presented here is a model defined on the kagome lattice, which has recently been found to display a Kondo-destruction quantum critical point[7]. Here, we consider the case when the energy dispersion in the non-interacting limit is shown in Fig. S7a, where the flat band is situated away from the Fermi energy. The dispersion for the interacting case ($u = 2.1$) is displayed in Fig. S7b, where once again, we observe the emergence of a flat band close to the Fermi energy. Due to the

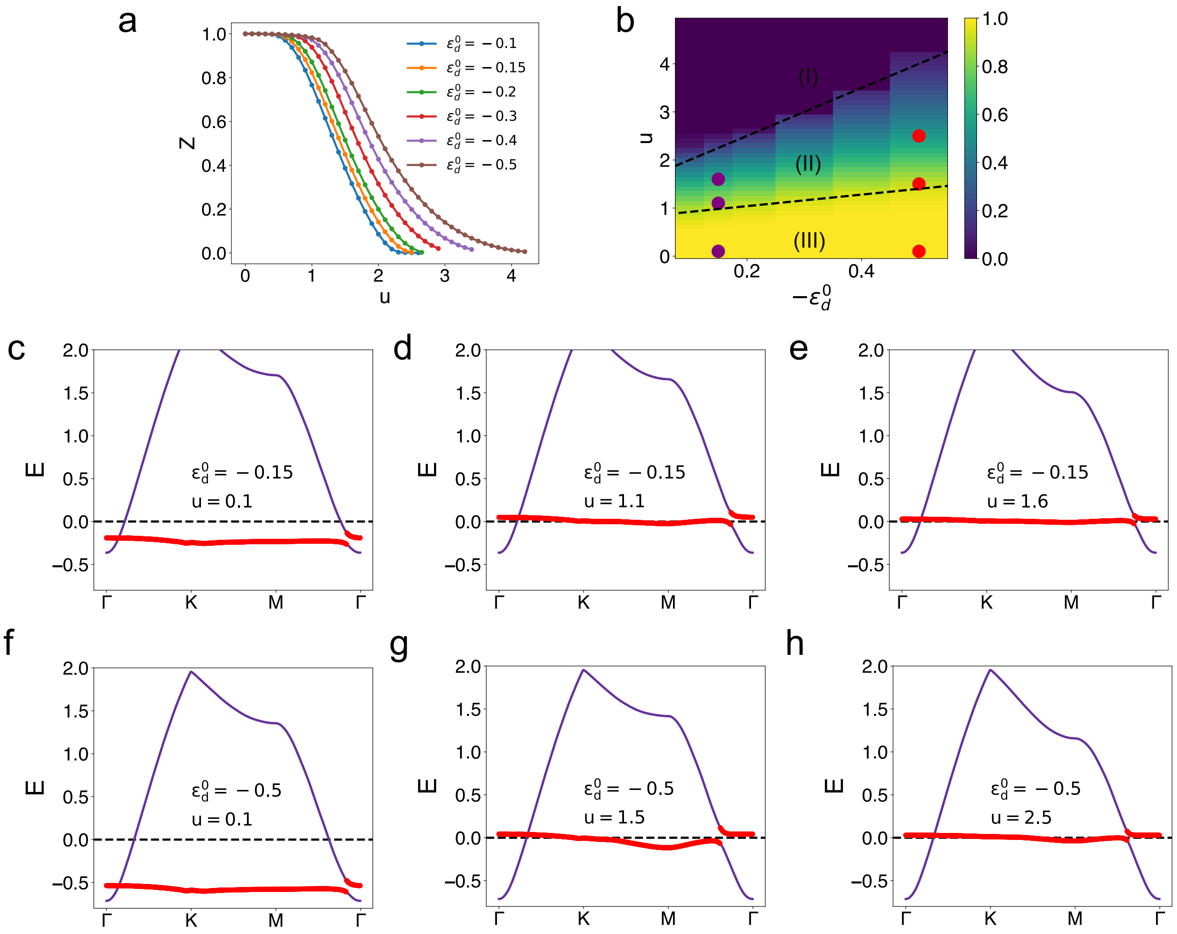

FIG. S6: **Phase diagram for the regime of emergent flat bands. a,** The quasiparticle weight of the more strongly correlated species of electrons (d) versus the interaction strength and for various ϵ_d^0. **b,** The corresponding colormap plot. In the parameter setting between the two black lines (region "II"), an emergent flat band develops in the immediate vicinity of the Fermi energy. **c-e,** The dispersion of the coherent single-electron excitations are shown for parameters corresponding to the purple points marked in **b**. **f-h.** The counterparts for the red points marked in **b**.

inclusion of spin-orbital coupling, the obtained solution is a topological insulator (TI), featuring a hybridization gap as depicted in the zoomed-in plot in Fig. S7c. Like in the canonical Kondo systems, the system is highly tunable. As shown in Fig. S7d, a relatively small Zeeman field leads to a substantial change in topology, where the TI gap is closed and nodal lines appear, which are protected by the M_z symmetry.

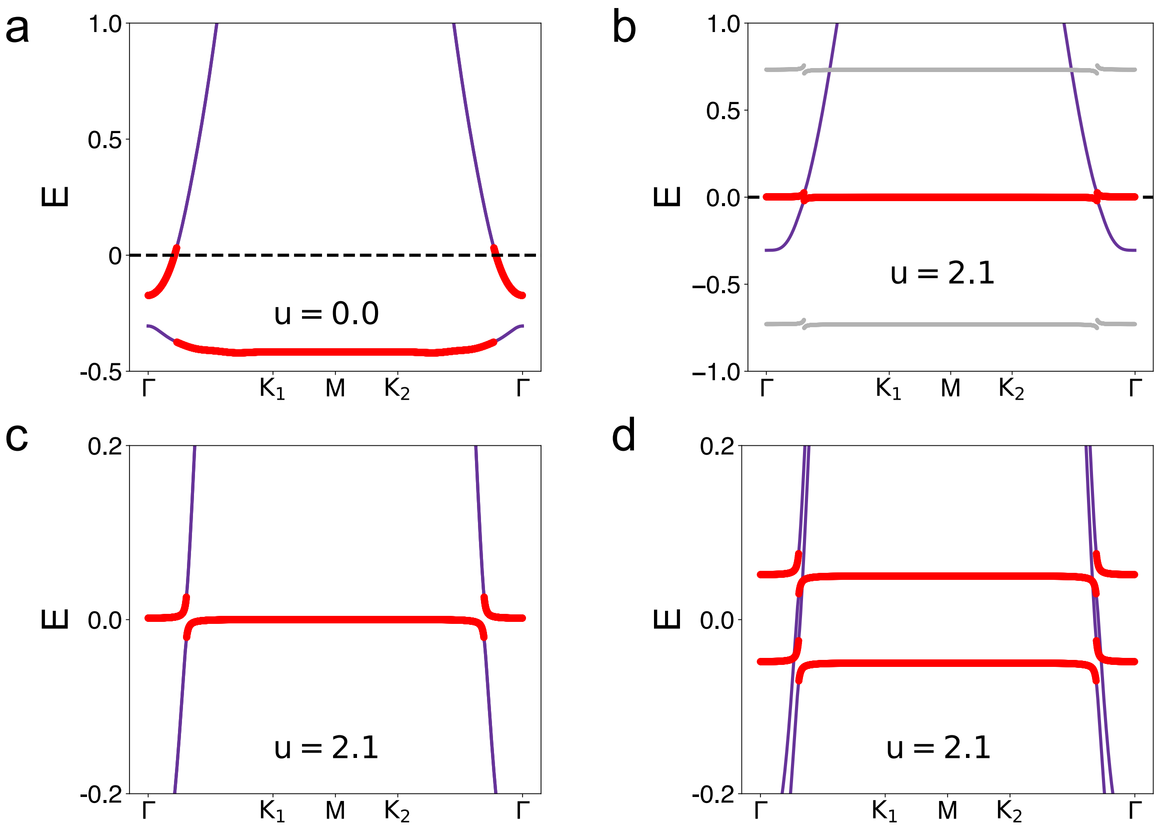

FIG. S7: **Topological Kondo effect in a general setting. a,** The noninteracting band structure. **b,** The dispersion of the coherent single-electron excitations at $u = 2.1$. The red solid curve denotes the emergent flat band close to the Fermi energy. The grey lines mark the incoherent single-electron excitations. **c,** The zoomed-in view of the emergent flat band. **d,** The band structure at $u = 2.1$ with a Zeeman splitting $m_z = 0.05$.

SUPPLEMENTARY REFERENCES

1. Wu, H., Chen, L., Malinowski, P., Jang, B. G., Deng, Q., Scott, K., Huang, J., Ruff, J. P. C., He, Y., Chen, X., Hu, C., Yue, Z., Oh, J. S., Teng, X., Guo, Y., Klemm, M., Shi, C., Shi, Y., Setty, C., Werner, T., Hashimoto, M., Lu, D., Yilmaz, T., Vescovo, E., Mo, S.-K., Fedorov, A., Denlinger, J. D., Xie, Y., Gao, B., Kono, J., Dai, P., Han, Y., Xu, X., Birgeneau, R. J., Zhu, J.-X., da Silva Neto, E. H., Wu, L., Chu, J.-H., Si, Q. & Yi, M. Reversible non-volatile electronic switching in a near-room-temperature van der waals ferromagnet. *Nat. Commun.* **15**, 2739 (2024).
2. Hu, H. & Si, Q. Coupled topological flat and wide bands: Quasiparticle formation and destruction. *Sci. Adv.* **9**, eadg0028 (2023).
3. Ding, W., Yu, R., Si, Q. & Abrahams, E. Effective exchange interactions for bad metals and implications for iron-based superconductors. *Phys. Rev. B* **100**, 235113 (2019).
4. Hu, H., Chen, L., Zhu, J.-X., Yu, R. & Si, Q. Orbital-selective mott phase as a dehybridization fixed point. *arXiv preprint arXiv:2203.06140* (2022).
5. Huang, J., Yu, R., Xu, Z., Zhu, J.-X., Oh, J. S., Jiang, Q., Wang, M., Wu, H., Chen, T., Denlinger, J. D., Mo, S.-K., Hashimoto, M., Michiardi, M., Pedersen, T. M., Gorovikov, S., Zhdanovich, S., Damascelli, A., Gu, G., Dai, P., Chu, J.-H., Lu, D., Si, Q., Birgeneau, R. J. & Yi, M. Correlation-driven electronic reconstruction in $FeTe_{1-x}Se_x$. *Commun. Phys.* **5**, 29 (2022).
6. Yu, R., Hu, H., Nica, E. M., Zhu, J.-X. & Si, Q. Orbital selectivity in electron correlations and superconducting pairing of iron-based superconductors. *Front. Phys.* **9**, 578347 (2021).
7. Chen, L., Xie, F., Sur, S., Hu, H., Paschen, S., Cano, J. & Si, Q. Metallic quantum criticality enabled by flat bands in a kagome lattice. *arXiv preprint arXiv:2307.09431* (2023).
